# Use of the repeated integral transformation method to describe the transport of solute in soil

**DOI:** 10.1016/j.heliyon.2022.e12774

**Published:** 2023-01-05

**Authors:** Elias Mwakilama, Duncan Gathungu, Vusi Magagula

**Affiliations:** a Dept. of Mathematics, Pan African University Institute for Basic Sciences Technology and Innovation (PAUSTI), Nairobi, Kenya; b Dept. of Pure & Applied Mathematics, Jomo Kenyatta University of Agriculture & Technology, Nairobi, Kenya; c Dept. of Mathematics, University of Eswatini, Kwaluseni, Matsapha, Eswatini; d Dept. of Mathematical Sciences, University of Malawi, Zomba, Malawi

**Keywords:** Solute, ADRE, Laplace, Fourier, Repeated integral transformation, Porous medium

## Abstract

Predicting the fate and transport of contaminants in soil or groundwater systems using analytical or numerical models is crucial for environmental researchers. While the analytical models are a flexible approach to quantifying the subsurface contamination and remediation because they are non-susceptible to numerical dispersions, economical and handier; two-dimensional analytical models that describe a bilateral flow coupled with both sink and decay factors are rarely reported. Motivated by the case of a non-bare soil ridge with constant point-solute source lying internally but parallel to the longitudinal flow direction, a (2 + 1) dimensional Advection-Diffusion-Reaction Equation (ADRE) of bilateral flow coupled with the linear sorption, decay, and sink is formulated to model the transport of dissolved solute in a homogenous and isotropic non-fractured porous medium. Then, a brief review of exact and analytical methods for solving the ADRE is conducted to establish the right solution methods. The Repeated Integral Transformation Method (RITM) is employed to derive the approximate analytical solutions for the formulated model, maintaining the model's original terms for the efficient sensitivity analysis. The RITM uses Laplace and Fourier transforms with a wide range of computed results. We compare the approximate solutions with numerical simulations in COMSOL to verify the accuracy of the approximate analytical models. Then, the application of the solutions is demonstrated through a systematic analysis of the effect on solute transport of advection-diffusion, reaction, sorption, retardation, sink, and pore water velocity. Results show that the presence of sink, mimicked by plant-root uptake activity, and decay tend to reduce the solute concentration in the medium. While both the retardation and sorption factors affect the movement of the dissolved solutes, water content and pore-water velocity promote the spreading of dissolved solutes. Solute concentration in the medium increases at low Peclet numbers, signifying the influence of diffusive coefficients. The current proposed RITM-based solutions can characterize contamination in the soil, and should be useful to environmental researchers.

## 1 Introduction

### 1.1 Background

The problem of studying solute transfer in a porous medium as directed by both fluid and soil properties [1–3] has received considerable experimental attention [4–6] because of its wide range of applications, for example, during the underground spread of pollutants [2,[7], [8], [9], [10]] or when monitoring water and solute flow [[11], [12], [13]]. Having a clear understanding of the mechanisms or dynamics of fate and transport of pollutants in soils and groundwater is therefore of particular interest for experimental and theoretical research in subsurface hydrology to sustain the environment. Mathematically, these dynamics can be described by a two-dimensional (2D) transport equation [[13], [14], [15], [16]],

1 $$u_{t}={\alpha_{x}u}_{xx}+{\alpha_{y}u}_{yy}+A\left(x,y\right)u_{x}+B\left(x,y\right)u_{y}+C\left(x,y\right)u+f\left(x,y,t\right)\text{,}$$

over the region Ω=[a,b]×[c,d]×[0,T) with initial (IC) and boundary conditions (BCs);

2 $$\begin{array}{ccc} u\left(x,y,0\right)=l\left(x,y\right)\text{,} & \left(x,y\right)\in \left(a,b\right)\times \left(c,d\right)\text{,} & \;\\ u\left(a,y,t\right)=g_{1}\left(y,t\right)\text{,} & u\left(b,y,t\right)=h_{1}\left(y,t\right)\text{,} & y\in \left(c,d\right),t>0\text{,}\\ u\left(x,c,t\right)=g_{2}\left(x,t\right)\text{,} & u\left(x,d,t\right)=h_{2}\left(x,t\right)\text{,} & x\in \left(a,b\right),t>0\text{,} \end{array}$$

where $u_{t}$ describes the temporal evolution of contaminant; $u_{xx}$ and $u_{yy}$ are spatial diffusive mechanisms, respectively; $u_{x}$ and $u_{y}$ are advective processes, respectively; while the last two terms represent the contaminate decaying factor and source position, respectively. Subject to the specific issue of address, solutions to the transport equation (Eqs. 1-2) can be analytically or semi-analytically derived [[17], [18], [19], [20], [21]]. On the other hand, numerical solutions may also be proposed [9,13,16,22]. Because most practically applied solute transport equations emanate from the deterministic governing equations such as Eqs. (1) and (2), several analytical or semi-analytical solutions remain valid. For example, other researchers use analytical solutions when conducting sensitivity analyses [5] or to investigate the effects of various parameters on the transport processes over large temporal and spatial scales where numerical solutions may be impractical [23]. In addition, the analytical solutions act as screening or benchmarking solutions for complex transport processes that cannot be exactly solved [6]. Unlike the numerical solutions, the analytical solutions lack susceptibility to numerical dispersion problems [2], are economical and handier [5]. In addition, others use analytical solutions to validate more comprehensive numerical solutions [24,25].

### 1.2 A rapid review of previous work on exact methods for solving equation (1-2)

Several methods of obtaining analytical solutions to a wide range of transport equations have been discussed [[26], [27], [28], [29]]. These include, separation-of-variables (SOV) [30,31] and the Green's Function Method (GFM) [32–35]. Although considered one of the oldest and most widely used techniques for solving transport equations, the SOV is limited to equations whose BCs are linear and homogeneous [30]. In addition, the SOV uses the assumption that the solution of the given equation is separable, that is, the solution to Eqs. (1) and (2) may be represented as u(x,y,t)=X(x)Y(y)T(t) where X, Y, and T are some functions dependent only on x, y, and t, respectively. This assumption, however, is often not met, and the process of separating a complex transport equation such as Eqs. (1) and (2) may not be easy [30].

In between the SOV method and numerical techniques for solving the transport equation, lies a family of transform methods [36] to which the similarity transformation method [22,37,38] based on group theory [[39], [40], [41]] belongs. The similarity (Boltzmann) transformation method involves changing the function u(x,y,t) space into a single ξ spatial variable [3,[42], [43], [44]]. This method successfully solves several but one-dimensional transport equations [3,38,45]. Another drawback of this method is identifying a suitable variable transformation [42]. On the other hand, the GFM belongs to the family of integral transforms. Because of its flexibility to handle arbitrary initial and boundary conditions and simplicity to represent solutions of multi-dimensional domains, the GFM has been widely used to solve various forms of solute transport equations [33,34,[46], [47], [48], [49], [50], [51], [52]]. The GFM, however, is more applicable to problems with source terms and inhomogeneous boundary conditions [9,29,53]. Nonetheless, integral-based analytical methods are generally attractive because they provide a simple evaluation of the analytical solution than methods that find the sum of infinite series [54].

Apart from the GFM, other integral-based methods [55,56] for solving transport equations are Laplace or Fourier transforms [[57], [58], [59]]. Commonly, the Laplace or Fourier integral transforms are independently applied [15,60,61] or are employed in combination with other non-integral methods [23,24,62] to solve simplified versions of Eqs. (1) and (2) type. Whenever the Laplace or Fourier transform is applied to transform Eqs. (1) and (2) from a real to a Laplace or a Fourier domain, sometimes the challenge is evaluating their inverses [63]. As a remedy, however, several numerical inverse Laplace [64] and inverse Fourier [65] algorithms have been developed and widely used [6,24,[66], [67], [68], [69]]. But, it is unlikely to get the solutions in an open or closed integrated form. Moreover, except for the works of Choudhury et al. [70], Moorthy [71], Abate et al. [72], and den Iseger [73], the majority of the available numerical inverse transform algorithms are only applicable to one-dimensional (1D) problems.

Since most transport equations in application exist in at least 2D, other researchers, e.g., Debnath and Dahiya [74], have extended the 1D Laplace transform to an n-dimensional Laplace transform, for n≥1. In this case, temporal and spatial variables are treated by the Laplace and its inverse in a 2-D problem, for example, for a function f(x,t). However, the challenge with this approach is finding the suitable inverse transform function in closed form when n>2. For example, owing to Shih et al. [75], more recently, Soko et al. [9] used the Fourier series numerical inverse algorithm [72] to evaluate the inverse of the 2D Laplace transform for a case of n=3 when f(x,y,t) was treated as a three-dimensional (3D) boundary value problem (BVP). Nonetheless, the solutions obtained via numerical inverse transforms, termed semi-analytical, are comparable to numerical solutions [24,62]. Alternatively, Kirkwood [35] suggests that to invert a multi-dimensional Laplace transformed equation, one needs to express it in Green's function form. The process, however, is not trivial.

To minimize the difficulties with inversion, other researchers suggest combining integral transforms with other methods. Guerrero et al. [23] combined the classical form of generalized integral transforms [76] with simple algebraic manipulations to derive an exact solution of the linear advection-dispersion (or diffusion) transport equation for both transient and steady-state regimes. Fityus and Smith [77] derived semi-analytic solutions to linearized 2D θ-based Richards' equation using repeated transforms (Laplace and Fourier transform) combined with Talbot's numerical inverse Laplace algorithm [78,79]. Gao et al. [6] first solved the solute transfer equation using Laplace transformation and applied de Hoog's [80] numerical inverse Laplace algorithm to obtain the end solution. Javidi et al. [62] combined Laplace transform with homotopy perturbation methods to solve Eqs. (1) and (2) and applied the Gaver-Stehfest's [81] numerical inverse Laplace algorithm to obtain the final solution. Dejam [24] solved the 1D advection-diffusion-reaction equation (ADRE) using Laplace transformation and applied the Fourier series inverse Laplace algorithm [82] to arrive at a final solution.

While the approach of combining integral transforms with other methods comes with several advantages, including simplifying derived solutions [6,62] or improving convergence [23,24], the resultant solutions still do not preserve model originality. For example, Guerrero et al. [23] first transformed the advection-diffusion equation into an exclusively diffusive problem. In Dejam [24], the 2D ADRE got reduced to an equivalent 1D advection-dispersive-reactive solute transport equation. Yates [83,84] and others observed that analytical inverse transforms are usually in multiple integrals which require numerical evaluations. Nonetheless, integral-based solutions preserve the nature of the model, thereby making it easy to use the derived analytical solutions [13].

Consequently, many studies solve transport equations using either Laplace or Fourier integral transforms or combining the two. For example, adapting from heat exchange analytical solutions of Carslaw and Jaegar [17] and Jost [85], Grisak and Pickens [5] combined Laplace and Fourier transforms to derive analytical solutions for advection-diffusive solute transfer equation in a fractured porous media. Similarly, Rahman et al. [4] solved two coupled 1D equations for solute transfer in a macropore-matrix domain using Laplace transformation. Genuchten and Alves [61] discuss exact, analytical, or approximate solutions to problems of 1D convective-dispersive equation (CDE) obtained using Laplace transform techniques. Chen et al. [86] derived solutions of a multidimensional Richards' equation for non-uniform distribution of rainfall intensity and arbitrary initial water content with a water table using the Fourier integral transform. Extending to previous work, Chen et al. [87] obtained convenient analytical solution for arbitrary surface fluxes before ponding using Fourier transforms on the linearized Richards’ equation. Javandel et al. [15] combined Laplace and Fourier transforms to derive various analytical solutions for a 2D solute transport in an infinite vertical aquifer with constant contaminant source lying orthogonal to the direction of groundwater flow. Shan and Javandel [25,60] extended the results of Javandel et al. [15] by deriving analytical solutions to the 2D advection-dispersion equation (ADE) with contaminant source lying parallel to the direction of groundwater flow in both finite and infinite domains using the Repeated Integral Transformation Method (RITM).

### 1.3 Motivation and objectives of this study

From the above rapid literature review, it clear that a variety of approaches to solving Eqs. (1) and (2) analytically are available, but the RITM is less employed. Such is the case despite some of its advantages. Unlike the GFM, the RITM offers an opportunity to represent part of the solution in closed form, thereby minimizing the number of numerical evaluations, pointed out by Yates [83,84]. In addition, like the GFM, the Generalized Integral Transform Technique (GITT) [88,89], and the Superposition principle method [90], the RITM is also capable of providing approximate solutions in finite, infinite, and semi-infinite domains [25,77]. Moreover, the RITM uses Laplace and Fourier transforms with minimal approximation errors [91]. Lastly, the RITM does not require a transformation of the original equation into a boundary value diffusion equation or inhomogeneous diffusion equation first [49,51,92], thereby maintaining all other model terms plus the advection, useful at sensitivity analysis stage.

Second, in agreement with Shan and Javandel [25], the majority of the reported analytical solutions for solute transport equations consider the source term f(x,y,t) to be either a point, a line, a plane, or a spatially distributed constant concentration or constant flux-rate of a known value on the surface of the porous medium but assumed lying perpendicular to the direction of flow [4,5,15,61,[93], [94], [95], [96]]. The analytical solutions presented by Shan and Javandel [25,60], in contrast to previous works, for a case of solute source lying parallel to flow direction, have a wide range of applications. However, like other previous studies, Shan and Javandel [25,60] considered the solute source to be located either at the top of the medium. Yet, a source of contamination to the medium may also be an internally located point, for example, solutes of fertilizer injected inside a ridge [97,98], but still regarded to be lying parallel to the direction of flow. Furthermore, Shan and Javandel [25,60] modeled a solute transport equation without the dispersion term in the direction of flow, thereby limiting the further application of the derived solutions. In addition, unlike with Javandel et al. [15], the “turbulent” advection term, perpendicular to the flow was not considered. Other researchers [[4], [5], [6],^24^] have also ignored either transverse advection or longitudinal diffusion processes when deriving analytical or semi-analytical solutions to Eqs. (1) and (2).

Motivated by the above discussions, this study has got three purposes. First, to model the transport of dissolved solutes from a constant source located internally but parallel to the longitude of the bilateral uniform flow. Second, to analytically solve the formulated model using the RITM. Third, to illustrate the application of the proposed solutions through a systematic analysis of the effects of advection, diffusion, sorption, retardation, sink, and pore water velocity on solute transport dynamics. The novelty in this case is both conceptual and methodological; because of, first, the manner we set-up a transport problem to illuminate a relevant topic further while relaxing the generalized bare surface assumption. Second, the way we apply the method of solution proven to work in other context for a 2 D bilateral flow case study.

Section 2 describes the formulation of the model. Section 3 presents approximate analytical solutions to the model. Simulation results to validate and analyze the solutions are presented and discussed in Section 4. Lastly, Section 5 provides a summary and the conclusions of the current study. Section 5 also highlights future study outlooks, derived from the current study's limitations. Guidelines leading to the derivations of the approximate analytical solutions are presented in the Appendix.

## 2 Mathematical formulation

### 2.1 Physical system

**Fig. 1:**
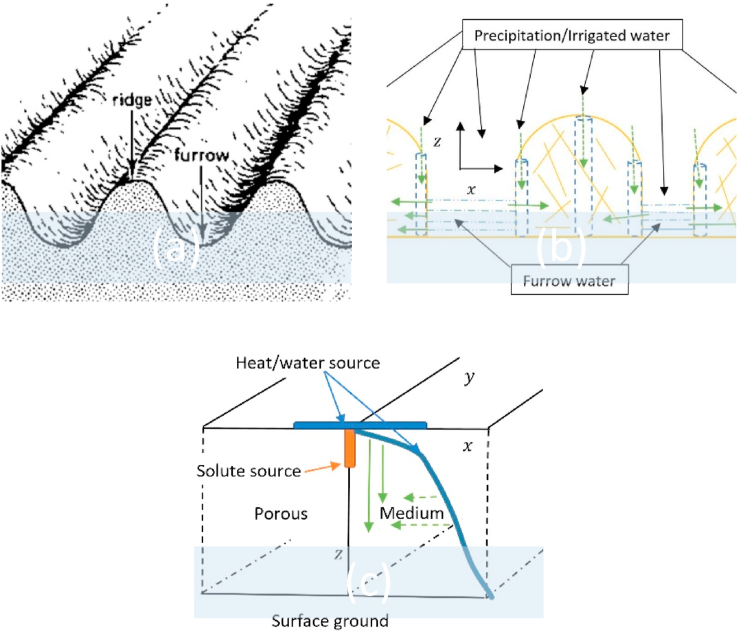
Geometry of the problem.

We consider the case of solute transport inside a homogenous and isotropic porous medium of ridge-furrow structure (Fig. 1(a)) with x, y, and z dimensions (Fig. 1(b)) but dominated by the steady uniform flow z-direction during infiltration as caused by precipitation. We assume that after a heavy precipitation, the furrows get filled with water which infiltrates horizontally along the x-direction (Fig. 1(c)). Unlike the previous studies [15,53,93,94,96], we consider a two-dimensional steady uniform flow field. Further, like in Shan and Javandel [25], Gao et al. [6], and Dejam [24], we assume dealing with a solute (fertilizer) that decays at a constant rate due to only first-order linear reaction factor.

**Fig. 2:**
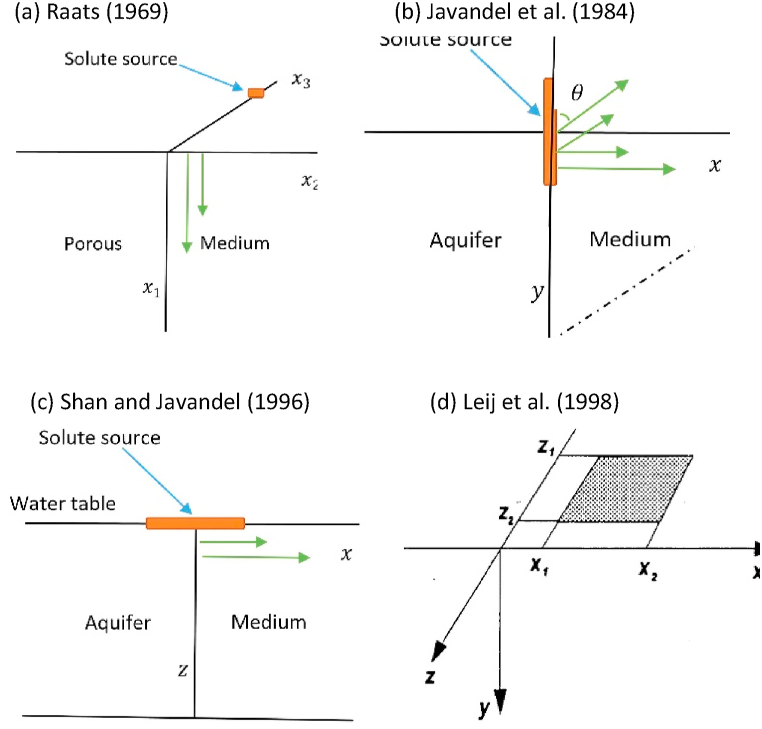
Descriptions of different solute source positions (a) point source, (b and c) line source, (d) planar source.

Hence, the motion of dissolved solutes as influenced by the movement of water is only modeled in planes parallel to the z and x. Although the geometry is similar to that of Noborio et al. [99] and Benjamin et al. [100] for numerically studying the transport of solute and moisture, respectively, we do not consider a bare surface. Moreover, even if Shan and Javandel [25] also considered the problem of a constant solute source lying parallel to the water flow direction, their formulated governing equation did not have the transverse advection and longitudinal dispersion/diffusion terms. Fig. 2 depicts various solute source positions as reported in the literature.

Like in Raats [101], the contaminant source is a point, however, located internally and parallel to the z-axis and perpendicular to the x-axis (see Fig. 1 (c)). The assumption of a constant solute source [15,25] is valid due to the study application [97,98]. On the other hand, although the assumption of a semi-infinite length in the z-direction can be somewhat restrictive [5], it has been demonstrated to be valid in the context where the concentration front does not reach the bottom of the domain [25]. It is also true for the case of a ridge-tillage system where for an injected fertilizer adjacent to the base of the plant [102,103], almost 95% percent of it is absorbed by the plant [97,98]. It is not the case for the bare ground where solute run-off process dominates. Unlike in groundwater systems considered by Javandel et al. [15], Shan and Javandel [25], Gao et al. [6], and others [21,61,83,104,105], mechanism of diffusion is more dominant in a ridge structure than dispersion.

### 2.2 Model

Based on the above conceptual description of the physical system, the model for reactive solute transport can generally be described by the advection-diffusion-reaction equation (ADRE) [24].

3 $$D\nabla^{2}C=\frac{\partial C}{\partial t}+\nabla ∙\left(\vec{u}C\right)+\lambda C\text{,}$$

where C(x,z,t) is the solute concentration [$mgL^{-1}$] at any point (x,z) and time t [h]; λ [−] is the decay constant; $\vec{u}$ is the average pore water velocity vector having components u and v [$mh^{-1}$] in the z and x directions, respectively; D is the mechanical dispersion coefficient with longitude $D_{L}=D_{e}+\alpha_{L}u$ [$m^{2}h^{-1}$] and transverse $D_{T}=D_{e}+\alpha_{T}v$ [$m^{2}h^{-1}$] components, respectively along with and perpendicular to the flow directions, where $D_{e}$ is the effective diffusion coefficient, while $\alpha_{L}$ and $\alpha_{T}$ [m] are longitudinal and transversal dispersions, respectively. In this study, like in the previous experimental and theoretical studies [4,24,46], both D and $\vec{u}$ are assumed to be scale-independent and linear.

Justified by an experimental study by Rahman et al. [4] for the transport of organic contaminants in soils, for example, fertilizer, Eq. (3) extends to include retardation $R_{m}$ [−], first-order sorption decay ${ή}_{w}\left[h^{-1}\right]$, and sink $\zeta_{s}$ [$h^{-1}$] factors. In addition, we assume that the volumetric water content $\vartheta_{w}$ [$m^{3}m^{-3}$] of the porous matrix and liquid diffusion gradient $D_{w}$ largely control the flux of solute-concentration coming from the source area into the soil matrix. Since the heat sourced from the surface of the ridge can influence the movement of the solute underneath, we describe the bilateral steady-state flow of solute transport as [[106], [107], [108]]

4 $$R_{m}\frac{\partial C}{\partial t}=\vartheta_{w}D_{w}\left(\frac{\partial^{2}C}{\partial x^{2}}+\frac{\partial^{2}C}{\partial z^{2}}\right)-D_{\eta }\left(\frac{\partial^{2}T}{\partial x^{2}}+\frac{\partial^{2}T}{\partial z^{2}}\right)-q_{w_{x}}\frac{\partial C}{\partial x}-q_{w_{z}}\frac{\partial C}{\partial z}-\left(\mu \vartheta_{w}C+{{ή}_{w}\kappa_{d}\rho }_{b}C_{a}\right)+\zeta_{s}C_{s}\text{,}$$

where $D_{\eta }$ [$m^{2}h^{-1}{{}^{\circ}C}^{-1}$] is the thermal gradient diffusion coefficient, T [°C] is the medium temperature, $q_{w_{x}}$ and $q_{w_{z}}$ are the flow rates [$mh^{-1}$] in x and z− directions, respectively; $\kappa_{d}$ [${mg}^{-1}L^{3}$] is the distribution coefficient for linear sorption process, μ [$h^{-1}$] is the first-order decay coefficient for degradation of solute in liquid phase, $\rho_{b}$ [$mgL^{-3}$] is the porous medium bulk density, and $C_{a}$ and $C_{s}$ are the solute concentrations at the adsorption (soil particles) and sink (plant root) sites, respectively. Alternatively, $\zeta_{s}$ can be defined as the first-order mass transfer coefficient [6].

The initial solute concentration $C_{0}$ is assumed to be zero everywhere in the domain, except at the source position. We consider that the initial amount of solute is close to the base of the plant, halfway between the width of the ridge, so that with the fact that v≪u, the concentration gradient ∂C/∂x at the x− boundaries is zero. Likewise, due to the influence of sink $\zeta_{s}$ factor, as caused by the plant root uptake, the amount of solute concentration at the infinite depth is also assumed negligible. It is worth noting that the assumptions of no-flow or zero-flux wall boundary conditions have also been used by Noborio et al. [13] and others [100,109] for the realistic analysis of the solute transport through a porous medium. Therefore, the initial condition (at t=0), and two sides (at x=±∞), top (at z=0), and bottom (at z=∞) boundary conditions are

5 $$\begin{array}{c} C\left(z,x,0\right)=C_{0}\\ C\left(0,x,t\right)=0\\ \lim_{x\to \pm \infty }\frac{\partial C}{\partial x}=0\\ C\left(\infty ,x,t\right)=0 \end{array}\}\text{.}$$

Introducing the following dimensionless variables:

6 $$x^{*}=\frac{x}{d},z^{*}=\frac{z}{L},\varphi =\frac{d}{L},T^{*}=\frac{\left(T-T_{0}\right)}{\left(T_{1}-T_{0}\right)},C^{*}=\frac{\left(C-C_{0}\right)}{\left(C_{1}-C_{0}\right)},t^{*}=\frac{u_{0}t}{d}\text{,}$$

where d, L, φ, $T_{1}$, and $C_{1}$ are the width size, depth size, aspect ratio, equilibrium temperature and uniform solute concentration of the medium, respectively, then Eq. (4) in the dimensionless form (upon dropping the asterisk) is

7 $$R_{m}\frac{\partial C}{\partial t}+{\varphi^{-1}q}_{w_{x}}\frac{\partial C}{\partial x}+{\varphi^{-1}q}_{w_{z}}\frac{\partial C}{\partial z}=\frac{\vartheta_{w}}{Pe}\left(\frac{\partial^{2}C}{\partial x^{2}}+\frac{\partial^{2}C}{\partial z^{2}}\right)-\Xi \left(\frac{\partial^{2}T}{\partial x^{2}}+\frac{\partial^{2}T}{\partial z^{2}}\right)-\varrho_{3}C-\varrho_{4}C_{s}+\varrho_{5}\text{,}$$

where Pe, and other coefficients $R_{m}$, $\vartheta_{w}$, Ξ , $\varrho_{3}$, $\varrho_{4}$, and $\varrho_{5}$ are the dimensionless Peclet number, and apparent retardation, volumetric water content, thermophoresis, decay, sorption, and sink parameters, respectively described as

8 $$Pe=\frac{u_{0}d}{D},\Xi =\frac{D_{T}}{T}\frac{T_{1}-T_{0}}{u_{0}d},\varrho_{3}=\frac{\mu \vartheta_{w}d}{u_{0}},\varrho_{4}=\frac{{ή}_{w}\kappa_{d}\rho_{b}d}{u_{0}\left(C_{1}-C_{0}\right)},\varrho_{5}=\frac{\zeta_{s}{dC}_{s}}{u_{0}\left(C_{1}-C_{0}\right)},R_{m}=1+\frac{\rho_{b}\kappa_{d}}{\vartheta_{w}}\text{.}$$

Because diffusivity may be inversely proportional to the viscosity of the medium [110], we further assume that $\vartheta_{w}$ is the total medium water content neglecting the fraction of water in the first few molecular layers of the surface that may exhibit high viscosity than that free water [111,112]. Making use of Eq. (6), the initial and boundary conditions, Eq. (5), in the dimensionless form (upon dropping the asterisk) are:

9 $$\begin{array}{c} C\left(z,x,0\right)=0\\ C\left(0,x,t\right)=1\\ \lim_{x\to \pm \infty }\frac{\partial C}{\partial x}=0\\ C\left(\infty ,x,t\right)=0 \end{array}\}\text{.}$$

### 2.3 Model re-arrangement

The dimensionless ADRE coupled with heat in the soil matrix, Eq. (7), is then re-arranged. First, recalling the assumption of a medium of low salinity with a non-bare surface, compared with Noborio et al.’s [99] and Benjamin et al.’s [100] study domains, it is assumed that the effect on the solute transport mechanism of heat is not significant. Thus, we can set the thermophoresis parameter Ξ to zero. Second, we re-arrange further the dimensionless parameters $\varrho_{3}$, $\varrho_{4}$, and $\varrho_{5}$. Introducing a “one” in the form of d/d to the right-hand side (RHS) of $\varrho_{3}$ in Eq. (8) leads to

10 $$\varrho_{3}=\frac{\mu \vartheta_{w}d^{2}}{Ď}\text{,}$$

where $Ď≅u_{0}d$ is the apparent diffusion of the porous matrix. Hence, Eq. (10) can be expressed in form of the Dank $\ddot{o}$ hler (Da) number [24]

11 $$Da=\frac{\kappa_{j}b^{2}}{D}\text{,}$$

when $\kappa_{j}\equiv \mu$, b≡d, and D≡Ď, where b, $\kappa_{j}\text{,}$ and D are half-fracture aperture length, reaction rate constant, and diffusion coefficient, respectively. In this study, we term the dimensionless parameter $\varrho_{3}$ (Eq. (10)) ‘apparent’ Dank $\ddot{o}$ hler number. In a similar manner, the re-arranged forms of $\varrho_{4}$, and $\varrho_{5}$ are

12 $$\varrho_{4}=\frac{\rho_{b}\kappa_{d}}{\left(C_{1}-C_{0}\right)}Da\;\text{and}\;\varrho_{5}=\frac{\zeta_{s}{d^{2}C}_{s}}{Ď\left(C_{1}-C_{0}\right)}\text{,}$$

respectively, where $\varrho_{4}$ in Eq. (12) is termed the ‘weighted’ Dank $\ddot{o}$ hler number.

Third, as demonstrated by Rahman et al. [4], the original retardation factor is expressed in terms of soil bulk density $\rho_{b\;}\left[mgL^{-3}\right]$; solutes distribution coefficient $\kappa_{d}\left[L^{3}{mg}^{-1}\right]$; and the volumetric water content $\theta_{w}\left[L^{3}L^{-3}\right]$ to yield ‘apparent’ retardation parameter $R_{m}^{\prime }=1+\rho_{b}\kappa_{d}/\theta_{w}$. Fourth, we use the linear relationship [113,114]

13 $$C_{a}=\kappa_{d}C_{aq}\text{,}$$

to re-arrange Eq. (7) where $C_{a}\;\left[mg{mg}^{-1}\right]$ is the amount of dissolved solutes sorbed at equilibrium aqueous concentration $C_{aq}\;\left[mgL^{-1}\right]$ [4]. Often, Eq. (13) means the units of $\kappa_{d}$ are expressed as $\left[L^{3}{mg}^{-1}\right]$ [115]. However, except for the expression of the retardation factor $R_{m}^{\prime }$, we assume $\kappa_{d}$ is dimensionless in a case where sorption takes place in an aqueous phase only because of the nature of the solute [114]. Hence, $C_{a}$ and $C_{aq}$ are of the same units $\left[mgL^{-1}\right]$ so that by substituting Eq. (13) into Eq. (7), we obtain

14 $$R_{m}^{\prime }\frac{\partial C}{\partial t}+{\varphi^{-1}q}_{w_{x}}\frac{\partial C}{\partial x}+{\varphi^{-1}q}_{w_{z}}\frac{\partial C}{\partial z}=\frac{\vartheta_{w}}{Pe}\left(\frac{\partial^{2}C}{\partial x^{2}}+\frac{\partial^{2}C}{\partial z^{2}}\right)-\left(\varrho_{3}^{\prime }+\varrho_{4}\right)C+\varrho_{5}^{\prime }\text{.}$$

Lastly, using the definition of the dimensionless Dank $\ddot{o}$ hler number (Da) again (Eq. (11)), $\varrho_{5}^{\prime }$ defined in Eq. (12) can be transformed further to get

15 $$\varrho_{5}^{\prime }=\frac{\zeta_{s}C_{s}L^{2}}{Ď\left(C_{1}-C_{0}\right)}\text{,}$$

where, as before, Ď is the apparent diffusivity of the porous medium.

## 3 Solution of the problem

Although expressed in its simplified form, it is evident that Eq. (14) still contains a large set of operator coefficients. Since these coefficients remain scale-independent, in compressed form, we consider the following equivalent terms: ${\lambda \equiv \varrho^{\prime }}_{3}+\varrho_{4}^{\prime }$, $R\equiv R_{m}^{\prime }$, $v_{x}={\varphi^{-1}q}_{w_{x}}$, $v_{x}={\varphi^{-1}q}_{w_{z}}$, and $\alpha_{x}=\alpha_{z}=\vartheta_{w}/Pe$ so that Eq. (14) is now expressed as [15]

16 $$R\frac{\partial C}{\partial t}+v_{x}\frac{\partial C}{\partial x}+v_{z}\frac{\partial C}{\partial z}=\alpha_{x}\frac{\partial^{2}C}{\partial x^{2}}+\alpha_{z}\frac{\partial^{2}C}{\partial z^{2}}-\lambda C+\varrho_{5}^{\prime }\text{.}$$

Due to the differences in the use of Eq. (14) compared with other equations in the case of constant solute concentration case parallel to flow direction [15,25], we solve Eq. (16) for two types of cases.

*3.1. Case I*: When $v_{x}=v_{z}=1.0$; R=1.0; $\varrho_{5}^{\prime }=0$; $-\lambda =\varrho_{3}+\varrho_{4}$; and $\alpha_{x}=\alpha_{z}=\frac{\vartheta_{w}}{Pe}$

Solving Eq. (16) subject to Eq. (9) using the RITM, yields [see the Appendix] the approximate solution

17 $$C\left(z,x,t\right)=\frac{\exp\left(-x\sqrt{\frac{\lambda }{\alpha_{x}}}\right)}{2}erfc\left(\frac{x}{2\sqrt{\alpha_{x}t}}-\sqrt{\lambda t}\right)\left\{1-\frac{1}{\sqrt{\lambda \alpha_{x}}}\right\}+\frac{\exp\left(x\sqrt{\frac{\lambda }{\alpha_{z}}}\right)}{2}erfc\left(\frac{x}{2\sqrt{\alpha_{x}t}}+\sqrt{\lambda t}\right)\left\{1+\frac{1}{\sqrt{\lambda \alpha_{x}}}\right\}+\int_{-\infty }^{\infty }\frac{1}{\pi {\tau^{\prime }}^{2}}\sqrt{\frac{\alpha_{x}}{2}}\left\{C^{k}\right\}d\tau^{\prime }+\frac{1}{2\alpha_{z}\sqrt{\pi \alpha_{x}}}\;\int_{0}^{t}\exp\left(\left(\frac{{-\left(x\right)}^{2}}{4\alpha_{x}\tau }\right)-\lambda \tau \right)\left\{erfc\left[\frac{\tau -z}{2\sqrt{\alpha_{z}\tau }}\right]\right\}\frac{d\tau }{\sqrt{\tau }}+\int_{-\infty }^{\infty }\frac{1}{\pi {\tau^{\prime }}^{2}}\sqrt{\frac{\alpha_{x}}{2}}\left\{C^{p}\right\}d\tau^{\prime }+\frac{1}{2\alpha_{z}\sqrt{\pi \alpha_{x}}}\;\int_{0}^{t}\exp\left(\left(\frac{{-\left(x\right)}^{2}}{4\alpha_{x}\tau }\right)-\lambda \tau \right)\left\{erfc\left[\frac{\left(z+1\right)-\tau }{2\sqrt{\alpha_{z}\tau }}\right]\right\}\frac{d\tau }{\sqrt{\tau }}\text{,}$$

where $C^{k}$ and $C^{p}$ in Eq. (17) are

18 $$C^{k}=\int_{0}^{\tau^{\prime }}\exp\left(\left(\frac{{-\left(x-\tau^{\prime }\right)}^{2}}{4\alpha_{x}\tau }\right)-\lambda \tau \right)\left\{erfc\left[\frac{\tau -z}{2\sqrt{\alpha_{z}\tau }}\right]\right\}\frac{d\tau }{\sqrt{\tau }}\;\text{,}$$

and

19 $$C^{p}=\int_{0}^{\tau^{\prime }}\exp\left(\left(\frac{{-\left(x-\tau^{\prime }\right)}^{2}}{4\alpha_{z}\tau }\right)-\lambda \tau \right)\left\{erfc\left[\frac{\left(z+1\right)-\tau }{2\sqrt{\alpha_{z}\tau }}\right]\right\}\frac{d\tau }{\sqrt{\tau }}\;\text{,}$$

respectively; erfc is the complementary error function, and τ and $\tau^{\prime }$ are the integral dummy variables in time and space, respectively. Now, since C(z,x,t) in Eq. (17) is a dimensionless quantity, we use the relative solute concentration

20 $$C^{*}=\frac{\left(C-C_{0}\right)}{\left(C_{1}-C_{0}\right)}\text{,}$$

to express the solution to the original equation, Eq. (7), as

21a $$C\left(z,x,t\right)=C_{0}+\left(C_{1}-C_{0}\right)\left[\frac{\exp\left(-x\sqrt{\frac{\lambda }{\alpha_{x}}}\right)}{2}erfc\left(\frac{x}{2\sqrt{\alpha_{x}t}}-\sqrt{\lambda t}\right)\left\{1-\frac{1}{\sqrt{\lambda \alpha_{x}}}\right\}+\frac{\exp\left(x\sqrt{\frac{\lambda }{\alpha_{x}}}\right)}{2}erfc\left(\frac{x}{2\sqrt{\alpha_{x}t}}+\sqrt{\lambda t}\right)\left\{1+\frac{1}{\sqrt{\lambda \alpha_{x}}}\right\}+\int_{-\infty }^{\infty }\frac{1}{\pi {\tau^{\prime }}^{2}}\sqrt{\frac{\alpha_{x}}{2}}\left\{C^{k}\right\}\;d\tau^{\prime }+\frac{1}{2\alpha_{z}\sqrt{\pi \alpha_{x}}}\;\int_{0}^{t}\exp\left(\left(\frac{{-\left(x\right)}^{2}}{4\alpha_{x}\tau }\right)-\lambda \tau \right)\left\{erfc\left[\frac{\tau -z}{2\sqrt{\alpha_{z}\tau }}\right]\right\}\frac{d\tau }{\sqrt{\tau }}+\int_{-\infty }^{\infty }\frac{1}{\pi {\tau^{\prime }}^{2}}\sqrt{\frac{\alpha_{x}}{2}}\left\{C^{p}\right\}d\tau^{\prime }+\frac{1}{2\alpha_{z}\sqrt{\pi \alpha_{x}}}\;\int_{0}^{t}\exp\left(\left(\frac{{-\left(x\right)}^{2}}{4\alpha_{x}\tau }\right)-\lambda \tau \right)\left\{erfc\left[\frac{\left(z+1\right)-\tau }{2\sqrt{\alpha_{z}\tau }}\right]\right\}\frac{d\tau }{\sqrt{\tau }}\right]\;\text{,}$$

where $C^{k}$, $C^{p}$, τ and $\tau^{\prime }$ are as defined before in Eq. 21a, 21b.

*3.2. Case II:* When $v_{x}\neq v_{z}\neq 1.0$; R≠1.0 ; $\varrho_{5}^{\prime }\neq 0$; $-\lambda =\varrho_{3}+\varrho_{4}$; and $\alpha_{x}=\alpha_{z}=\frac{\vartheta_{w}}{Pe}$

In a similar manner, using the RITM, we obtain the approximate analytical solution

22 C(z,x,t)={1−vzλαz}(exp(−zλαz)2erfc(z2αztR−λtR)−exp(zλαz)2erfc(z2αztR+λtR))+ϱ5′2λαz∫0tR(exp(−(z−τ)λαz)erfc(−(z−τ)2αztR+λtR)−exp((z−τ)λαz)erfc((z−τ)2αztR+λtR))dτ+∫−∞∞δ(z)πτ′2αz2{∫0τ′Rexp((−(z−τ′)24αzτ)−λτ)erfc[vxτ−x2αxτ]dττ}dτ′+vz2αxπαz∫0tRexp((−(z)24αzτ)−λτ)erfc[vxτ−z2αxτ]dττ−∫−∞∞ϱ5′δ(t)2αxπαz{∫0τ′Rexp((−(z−τ′)24αzτ)−λτ)erfc[vxτ−x2αxτ]dττ}dτ′+∫−∞∞δ(z)πτ′2αz2{∫0τ′Rexp((−(z−τ′)24αzτ)−λτ)erfc[(x+1)−vxτ2αxτ]dττ}dτ′+vz2αxπαz∫0tRexp((−(z)24αzτ)−λτ)erfc[(x+1)−vxτ2αxτ]dττ−∫−∞∞ϱ5′δ(t)2αxπαz{∫0τ′Rexp((−(z−τ′)24αzτ)−λτ)erfc[(x+1)−vxτ2αxτ]dττ}dτ′,

where δ(u) is the Dirac delta function. In this study, we consider δ(u) defined by [59]

21b δ(u)={0,u≤01U,0<u≤U0,u>U.

Guidelines leading to the derivation of the approximate analytical solutions (Eqs. (17) and Eq. (22)) to the model (Eq. (16)) with the conditions (Eq. (9)) are presented in the Appendix. We evaluate these solutions through a systematic analysis of the effect of model parameters on the solute distribution in the next section using parameter values taken from the literature. The approximate analytical models are evaluated numerically using the Algorithm (1) as described below.

**Algorithm 1:** Psuedocode of Algorithm for Analytical Computations

1:	**Start procedure** OUTPUT = MAIN FUNCTION (INPUTS)
2:	Set all physical parameters;
3:	Set solution matrix;
4:	$t=linspace\left(t_{0},t_{f},N\right)$; ← define time space
5:	$N∶=N_{\max}$; ← define upper limit for time integrals
6:	**Loop:**
7:	varying parameter selection
8:	Figure;
9:	**For**j=1:length(parameterset)**do**
10:	Set up coefficients;
11:	**Loop:**
12:	**For**$i=1:N_{\max}$**do**
13:	Trap−fun; ← evaluate time integral sub-functions using Trapezoidal rule algorithm
14:	Spac−fun; ← evaluate space integral sub-functions using Gauss-Krourod quadrature method
15:	**Return**Output(i)∶=function(Aufun,Trap−fun,Spac−fun)
16:	**Endfor**
17:	Plot figure ← for each j
18:	**Goto**j− loop
19:	**Endfor**;
20:	Pf∶=Aufun(inputs)← define auxiliary sub-function; **return**Pf;
21:	**End procedure**

## 4 Results and discussion

### 4.1 Model verification

**Fig. 3:**
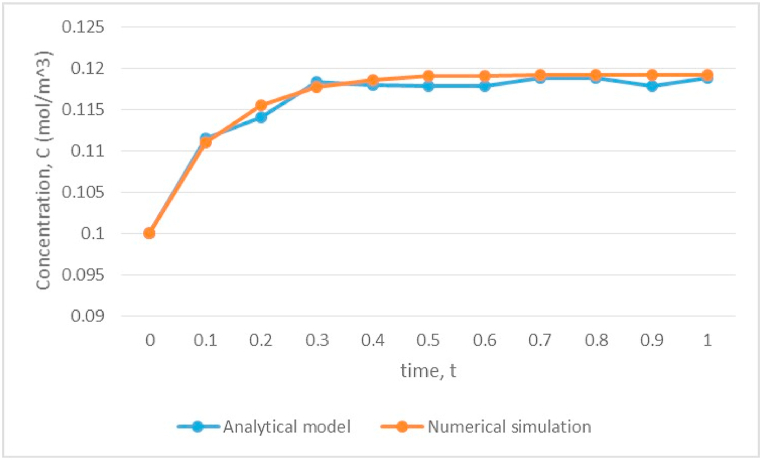
Verification of the developed analytical model using a numerical simulation.

The approximate analytical models developed here consider the case of modelling solute transport inside a homogenous and isotropic porous medium of ridge-furrow structure with steady bilateral groundwater flow under constant-point solute source. To verify the accuracy of the approximations, the approximate analytical solution, Eq. (17), is compared with a numerical solution of the original 2D governing equations, Eq. (16) with the conditions Eq. (9). Following the structure of Eqs. (16) and (9), the numerical simulation is performed using the Coefficient form PDE module (finite element based technique) in COMSOL Multiphysics commercial software [116,117]. In COMSOL, the Coefficient form PDE is described as

23 $$e_{a}\frac{\partial^{2}u}{\partial t^{2}}+d_{a}\frac{\partial u}{\partial t}+\nabla ∙\left(-c\nabla u-\alpha u+\gamma \right)+\beta ∙\nabla u+au=f\text{,}$$

where ∇=∂/∂x, $e_{a}$ is the mass coefficient, $d_{a}$ is the damping coefficient, c is the diffusion coefficient, α is the conservative flux, γ is the conservative flux source, β is the convection coefficient, a is the absorption coefficient, and f is the source term. Hence, the model, Eq. (16), is numerically solved in COMSOL via the transformation

24 $$\left(\begin{array}{cc} R & 0\\ 0 & 0 \end{array}\right)\left[\begin{array}{c} \frac{\partial u_{1}}{\partial t}\\ \frac{\partial u_{2}}{\partial t} \end{array}\right]+\frac{\partial }{\partial x}\left(\begin{array}{c} -\alpha_{x}\frac{\partial u_{1}}{\partial x}\\ 0 \end{array}\right)+\frac{\partial }{\partial z}\left(\begin{array}{c} -\alpha_{z}\frac{\partial u_{1}}{\partial z}\\ 0 \end{array}\right)+v_{x}\left(\begin{array}{c} \frac{\partial u_{1}}{\partial x}\\ 0 \end{array}\right)+v_{z}\left(\begin{array}{c} \frac{\partial u_{1}}{\partial z}\\ 0 \end{array}\right)=\left(\begin{array}{c} \varrho_{5}^{\prime }-\lambda u_{1}\\ 0 \end{array}\right)\text{,}$$

where $e_{a}=0=\gamma =\alpha$, $u_{1}\equiv C$, $\alpha_{x}=\alpha_{z}\equiv c$, and $\beta \equiv v_{x}=v_{z}$. Together with the condition values, Eq. (9), the verification is done at parameter values $v_{x}=1=v_{z}$, $\kappa_{d}=0.013$, $\rho_{b}=1700$, $\theta_{w}=0.42$, λ=0.1, and Pe=0.30. In COMSOL, the time step is chosen as Δt=0.1 and the numerical simulation started from t=0 and ended at t=1.0 under a fine grid mesh (triangular mesh consisting of 1326 elements, with a minimum quality of 0.6684 and an average quality of 0.9095) on rectangular domain. Results are shown in Fig. 3.

According to Fig. 3, there is a good agreement between the developed analytical model and the results of the numerical simulation, particularly at the beginning time of the simulation. Therefore, the proposed analytical model is verified, rendering it possible for modeling the distribution of solute transport. Then, the systematic analyses of the effect of varying the model parameters on the solute concentration profiles were carried out to validate the efficacy of the proposed approximate analytical solutions.

### 4.2 Model analysis

To analyze the model and demonstrate the application of the approximate analytical solutions, we considered various critical model parameters of flow and solute transport in porous media [118] including advection and diffusion, retardation, sorption, chemical reaction, porosity, sink, and pore water velocity. Such analysis is pertinent to the formulation and validation of solute transport models [5,6,22,24] and relevant to water and chemical management studies. Moreover, systematic analyses are needed to predict the movement of agricultural chemicals through the soils. Many researchers such as Rahman et al. [4], Grisak and Pickens [5], Gao et al. [6], Dejam [24], and Zhou and Wang [119] have also considered this approach of validating models. However, the majority simplify the analysis by ignoring the effects of one thing over the other. For example, Gao et al. [6] didn't consider the sorption and degradation of solute and the molecular diffusion. In addition, they conducted most of the analyses on 1D fractured porous soil columns. The present analytical solutions permit the investigation of the effects of several parameters on the solute transport in a 2D non-fractured porous media. We set the aperture ratio of the medium system φ to 0.42. We take other parameter values from the experimental studies of *Griffioen* et al. [120], Zhou and Wang [119], and Rahman et al. [4], and other values as used by Gao et al. [6] and Dejam [24].

(a) Effects of advection and diffusion on breakthrough curves

**Fig. 4:**
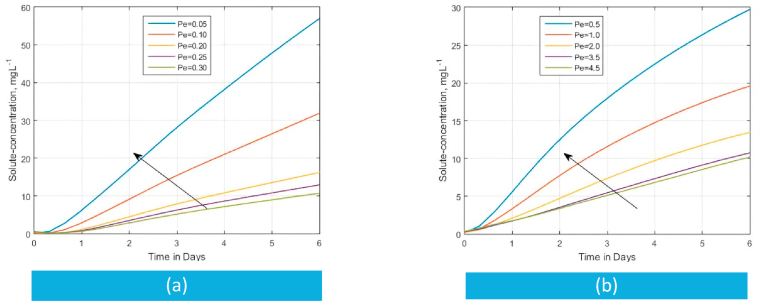
The role of the Peclet number Pe on the solute concentration within a semi-infinite matrix domain versus time for the derived analytical model at $\vartheta_{w}$ = 0.20, $R_{m}=1.0$, $\alpha_{x}=\alpha_{z}=\vartheta_{w}/Pe$ , $q_{w_{x}}=q_{w_{z}}=1.0\text{,}$$C_{0}=0.1$ and $C_{1}=0.125$

Among several uses [[121], [122], [123], [124]], the Peclet number Pe relates the effect of advection and diffusion on solute transfer curves by providing a measure of the movement of the solute by mass flow [119]. Dejam [24] reported that increasing Pe entails solute breakthrough because of the higher advection than the diffusion process. To the contrary, a small Pe (e.g., Pe<0.3) value reflects greater diffusivity where advection is very slow, and molecular diffusion almost controls the dispersion ($Pe=u_{0}d/D$) completely [24,125]. The effect of the magnitude of diffusion coefficient $\alpha_{x}$ on the BTCs is illustrated by the changes in values of Peclet numbers in Fig. 4 (a) and Fig. 4 (b). Increasing Pe values results in a significant effect on the diffusion process of solute concentration. In agreement with Dejam [24] and others [124], the fast break-through resulting from increasing Peclet numbers is due to the advection process which causes a low soil matrix concentration state in the medium. Instead, we observe that the soil matrix has an increased concentration state at low Peclet numbers (Fig. 4) for a long distribution time. The reason is that at small Pe values, the diffusion mechanism is more dominant than advection.

On the other hand, when we choose Peclet numbers from the transition regime (0.3<Pe<5), where the advection process contributes more to the dispersion [24,125], distributions of solute concentration are affected (Fig. 4 (b)). This is the case because increasing Pe enhances the effect of dispersion due to an increase in advection. Since a large Pe implies that convection is more pronounced than diffusion [121], the solute concentration moves due to flow rather than diffusion. Consequently, as evidenced by the distances between low-Pe curves and high-Pe curves, a wide range of concentration distribution is seen in Fig. 4(b) than before (Fig. 4(a)) due to the influence of hydrodynamic dispersion over diffusion [123].

(a) Effect of chemical reaction on breakthrough curves

Previously, researchers [6,22,24,[126], [127], [128]] investigated the effect of chemical reaction on heat or mass transfer using different but related forms of chemical reaction parameters. To cite a few, Ravindran et al. [22] used a chemical reaction parameter $\Delta =k_{c}/\nu$, where $k_{c}$ and ν were respectively, defined as chemical reaction rate and kinematic viscosity. Similarly, Sharma and Konwar [126] considered a chemical reaction parameter $\gamma =k_{c}/\Omega$, where Ω is the uniform angular velocity. In its simplicity, Gao et al. [6] used a constant linear mass transfer coefficient ω as a chemical reaction parameter. Anwar et al. [127] considered $R=R_{0}/a$ a chemical reaction parameter, where $R_{0}$ and a were a chemical reaction coefficient and a constant coefficient of a linear scale-dependent stretching velocity component. However, Dejam [24] used the dimensionless Dank $\ddot{o}$ hler (Da) number (Eq. (11)) to describe the influence of chemical reaction parameter. Since the Dank $\ddot{o}$ hler (Da) number relates diffusion to reaction processes [24], we study the effect of chemical reaction on BTCs using the ‘apparent’ Dank $\ddot{o}$ hler number $\varrho_{3}=\vartheta_{w}Da$ (Eq. (10)) that contains both the volumetric water content $\vartheta_{w}$ and the first-order linear decay rate μ.

**Fig. 5:**
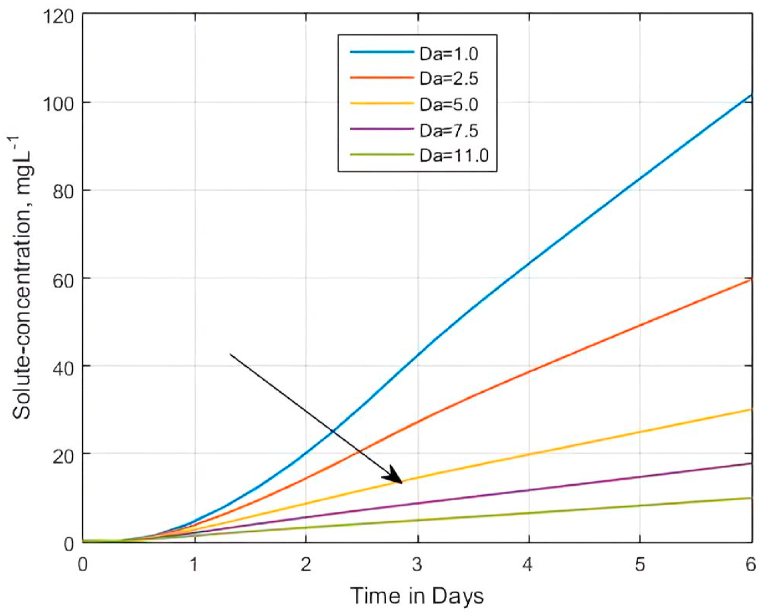
Effect of apparent Dank $\ddot{o}$ hler number ${Da}^{\prime }$ on the solute concentration verses time for the derived analytical solution when Pe=0.10, $\vartheta_{w}$ = 0.20, $R_{m}=1.0$, $\alpha_{x}=\alpha_{z}=\vartheta_{w}/Pe$ , $q_{w_{x}}=q_{w_{z}}=1\text{,}$$C_{0}=0.1$ and $C_{1}=0.125$ in the porous medium.

Dejam [24] reported that increasing the rate of reaction (or the Dank $\ddot{o}$ hler (Da) number) affects the breakthrough of solutes much more in the ‘fracture/pore’ than in the soil matrix [24]. We had anticipated that the influence of reaction rate on BTCs in the non-fractured porous matrix would be insignificant. Thus Da in $\varrho_{3}$ would have had a contrary effect on the BTCs than Pe in the advection-diffusion case. However, in agreement with Dejam [24], Ravindran et al. [22], and Anwar et al. [127], Fig. 5 shows that μ has a significant effect on the solute BTCs. In particular, the solute-concentration state or the BTC in a 2.5 m × 2.5 m domain described by Eq. 21a, 21b decreases/slows down as Da increases, consistent with Dejam [24]. The results signify the role played by μ in the model. The explanation is that with an increase in Da, the decay rate μ increases, resulting in the generation of more species [22]. The latter process enhances the mass transfer rate, thereby causing the concentration state of the fluid to decrease.

Further, Fig. 5 illustrates that when Da decreases, by its definition, the influence of diffusion is more pronounced than before as the solute concentration in the medium increases. Unlike in Dejam [24], the current deduced apparent Dank $\ddot{o}$ hler number $\varrho_{3}$ goes an extra mile by relating the diffusion, reaction, and volumetric water content $\vartheta_{w}$ terms. Because of that, we also investigated the contribution of $\vartheta_{w}$ towards studying the effect of chemical reactions on the solute BTCs.

**Fig. 6:**
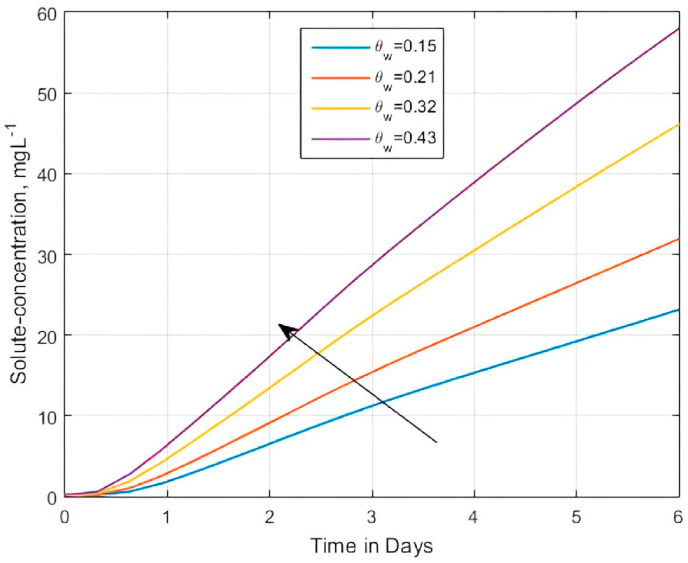
The impact of $\vartheta_{w}$ on the solute transfer within the semi-infinite matrix domain at 2.5 m. The expression of diffusion-reaction dependent diffusivity used in Eqn. (20) is $\varrho_{3}={Da}^{\prime }=\vartheta_{w}Da$ for $\alpha_{x}=\alpha_{z}=\vartheta_{w}/Pe$ when $Pe=0.25,q_{w_{x}}=q_{w_{z}}=1.0\text{,}$$C_{0}=0.1$ and $C_{1}=0.125$

Described as the volume wetness or mass wetness, soil water content $\vartheta_{w}=\left(\rho_{b}/\rho_{w}\right)m_{w}$ measures the amount of water (volume or mass) contained in a unit volume or mass of soil [129]. We, therefore investigated the potential effect of the volumetric water content $\vartheta_{w}$ on accelerating solute solubility and transport within the semi-infinite soil matrix for the developed model using Eq. (17) for different values of $\vartheta_{w}$ ($\vartheta_{w}$ = 0.05, 0.1, 0.2, and 0.3) at a fixed value of Da (Da = 2.0). As expected, Fig. 6 suggests that an increased solute concentration state results in a higher moisture content, which according to Ratts [101], enhances the solubility of solutes [130] and chemical reaction [131]. The ‘apparent’ Dank $\ddot{o}$ hler number $\varrho_{3}=\vartheta_{w}Da$ within the matrix domain should account for the results in Fig. 6, where $\vartheta_{w}$ acts as an accelerator for the movement of solute concentration by diffusion process from a region of high concentration to that of a low concentration. Consequently, this process creates a negative concentration gradient, thereby increasing the mass transfer coefficient. In turn, the concentrates of the medium state increase also. Thus, Fig. 6 suggests that the concentration of the medium state increases as $\vartheta_{w}$ increases. The results are in agreement with the experimental studies of Jungk and Claassen [110] and Bhadoria et al. [132] that showed that, when expressed as a function of the volumetric soil water content $\vartheta_{w}$ [133], effective diffusion $D_{e}$ ($D_{e}=D_{e}\vartheta_{w}f\left(1/b\right)$) of phosphate (P) and potassium (K) increased by a factor of 25% with an increase in water content.

(b) Effect of sorption and retardation on breakthrough curves

According to Núñez-Delgado et al. [134], sorption is the process by which a substance (sorbent) is sorbed (adsorbed or absorbed) on or in another (sorbent). Under unsaturated conditions, an increase in the sorption of solute particles to soil particles limits movement of the contaminant inside the porous medium [135]. In the current study, we investigated the role of the linear isotherm sorption process $\kappa_{d}$ on the solute concentration within the porous matrix for the developed model using Eq. 21a, 21b in two different ways. We first considered the influence of $\kappa_{d}$ within the retardation factor $R_{m}$ on the distribution of contaminants because, in the model, the retardation factor accounts for linear, instantaneous, and reversible equilibrium sorption of reactive solutes [61,123]. Secondly, we investigated the effect of sorption on solute breakthrough curves using the interaction means of $\kappa_{d}$ and ${ή}_{w}$ inside the sorption parameter $\varrho_{4}$, herein referred to as the ‘weighted’ Dank $\ddot{o}$ hler number. These investigations were done because solute concentration movement can be retarded due to the physical restrictions imposed by the sorption mechanism in partially saturated porous media.

**Fig. 7:**
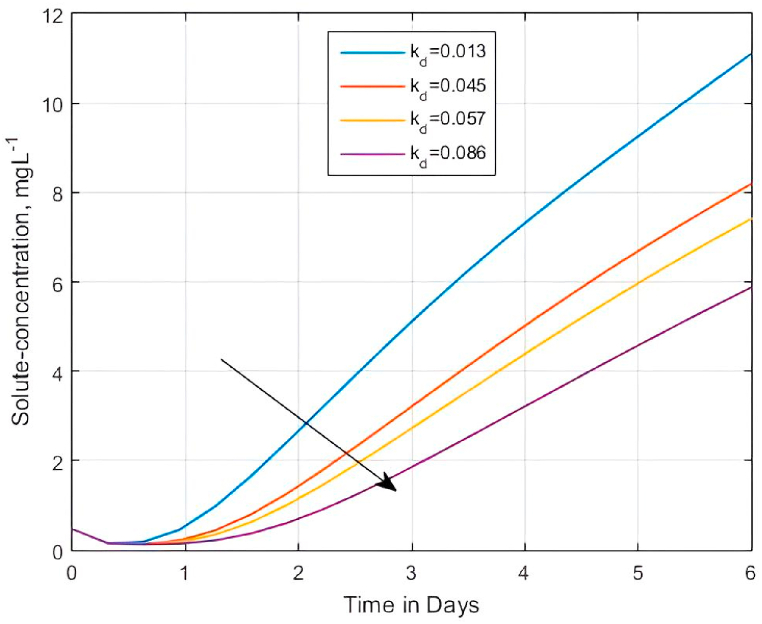
Effect of the retardation factor $R_{m}$ on solute concentration profiles for $R_{m}=1+\left(\rho_{b}\kappa_{d}\right)/\vartheta_{w}$ when $\kappa_{d}$ = (0.013, 0.045, 0.057, and 0.086), $\vartheta_{w}$ = 0.21, Pe=0.25, $\alpha_{x}=\alpha_{z}=\vartheta_{w}/Pe$, $q_{w_{x}}=q_{w_{z}}=1.0,{\;\rho }_{b}=2.69\;g{cm}^{-3}$, $C_{0}=0.1$ and $C_{1}=0.125$

For the case of the effect of retardation ($R_{m}$) on BTCs, increasing $\kappa_{d}$ or decreasing $\vartheta_{w}$ may influence the decrease of diffusion process due to an increase in the retardation factor. Therefore, $\vartheta_{w}$ may cause a dissimilar effect on the BTCs, unlike in the case of a reaction process. Nonetheless, what is central in this sorption case is the role of linear sorption or the distribution coefficient $\kappa_{d}$. Unlike in non-sorbing solutes, sorbing solutes contribute more to the growth of the retardation process [4]. Fig. 7 shows that the BTCs at 2.5 m described by Eq. 21a, 21b decrease with increasing values of $\kappa_{d}$ ($\kappa_{d}$ = 0.013, 0.045, 0.057, and 0.086). Increasing the $\kappa_{d}$ influences the solute BTCs to take long breakthrough times since the adsorptive molecules are held back by the adsorbent [118].

**Fig. 8:**
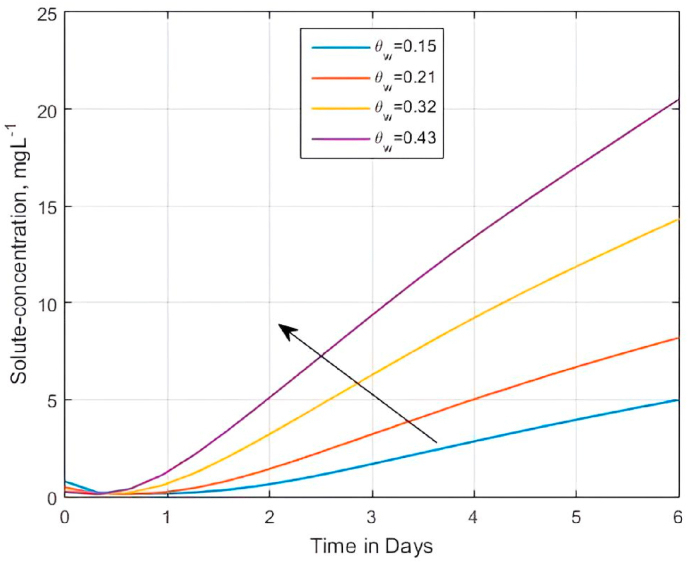
The role of volumetric water content as porosity on the influence of retardation factor on solute concentration profiles within the matrix versus time for $R_{m}=1+\left(\rho_{b}\kappa_{d}\right)/\vartheta_{w}$,when $\alpha_{x}=\alpha_{z}=\vartheta_{w}/Pe$ , Pe=0.25,$q_{w_{x}}=q_{w_{z}}=1.0\text{,}$$C_{0}=0.1$ and $C_{1}=0.125$

Alternatively, with increased values of $\vartheta_{w}$ ($\vartheta_{w}$ = 0.05, 0.1, 0.2, and 0.3) at a fixed value of $\kappa_{d}$ ($\kappa_{d}$ = 0.013 ${cm}^{3}g^{-1}$), Fig. 8 shows that the solute concentration profiles diffuse highly at increased values of $\vartheta_{w}$ because, when $\vartheta_{w}$ is porosity inside a retardation factor $R_{m}=1+\rho_{b}\kappa_{d}/\vartheta_{w}$, the soil becomes more permeable, thereby reducing the resistance to flow factor $R_{m}$. Moreover, according to Voroney [136], the volumetric soil water content at the saturation point is equivalent to the total soil pore space. Henceforth, consistent with [132], the results show that the concentration state increases with an increase in $\vartheta_{w}$ and a corresponding decrease in $R_{m}$. The chosen parameter values are about the experimental study of Rahman et al. [4] and correspond with a non-equilibrium sorption condition inside the matrix region. For the simulation results appearing in Figs. 7 and 8), we used the value of Pe=0.25 corresponding to the range ${1<R}_{m}\leq 1.3$ computed by the graphical method [123].

**Fig. 9:**
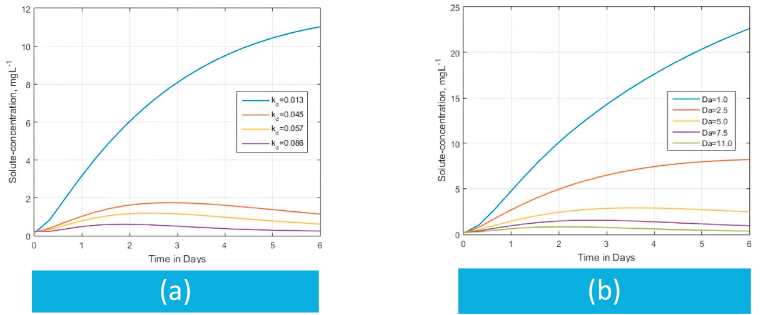
The impacts of the distribution coefficient $\kappa_{d}$ and Dank $\ddot{o}$ hler Da numbers in the decaying factor λ within the porous matrix on the dimensionless solute concentration profiles versus time for the developed model using Eqn. (11) whenever $\rho_{b}=2.69\;g{cm}^{-3}\text{,}$$C_{0}=0.10\;g{cm}^{-3}$; $C_{1}=0.125\;g{cm}^{-3}$, $\vartheta_{w}$ = 0.21, $R_{m}=1$, $q_{w_{x}}=q_{w_{z}}=1.0\text{,}$ and Pe = 0.25z.

Since the decaying factor, λ in Eq. (16), is the sum $\left(\varrho_{3}+\varrho_{4}\right)$, we assumed $\varrho_{3}=0$ so that $\lambda =\varrho_{4}$ and investigated the role of $\varrho_{4}$ on solute distribution. Thus, an expression for $\varrho_{4}=\left(\rho_{b}\kappa_{d}Da\right)/\left(C_{1}-C_{0}\right)$ was used to replace λ. Fig. 9 shows the results of solute distribution profiles due to the impact of $\kappa_{d}$ ($\kappa_{d}$ = 0.013, 0.045, 0.057, and 0.086) at a fixed value of Da (Da = 2.0) (Fig. 9 (a)). On the other hand, the impact of first-order reaction rate Da (Da = 1, 2, 5, 7, and 10) at a fixed value of $\kappa_{d}$ ($\kappa_{d}$ = 0.013) is shown in (Fig. 9 (b)). Fig. 9 (a) indicates that the solute distribution profile increases at the lowest value of $\kappa_{d}$. Consistent with Rahman et al. [4], the levels of solute concentration state decrease for a corresponding increase in $\kappa_{d}$ because values of $\kappa_{d}$ largely influence the decaying process. Consequently, there is a high degree to which a contaminant is sorbed by the sorbent causing the solute concentration state in the medium to decrease [135]. However, the shapes of the curves (Fig. 9 (a)) suggest that the nonlinear sorption isotherm $S=k_{f}C_{w}^{n}$ for n<1 where $k_{f}$ is the Freundlich sorption coefficient could have been an appropriate assumption during model formulation. We leave it for future investigations.

Alternatively, when the increase in the ${ή}_{w}$ described by Da influences the decaying factor λ, we observe that the concentration state of the fluid decreases with an increase in ${ή}_{w}$, consistent with other studies [6,22,24]. The reason is that an increase in ${ή}_{w}$ should influence the increase in specie generation, thereby increasing the mass transfer coefficient λ [22], causing a decrease in the concentration state and the concentration boundary layer of the porous medium.

(c) Effect of sink coefficient on breakthrough curves

**Fig. 10:**
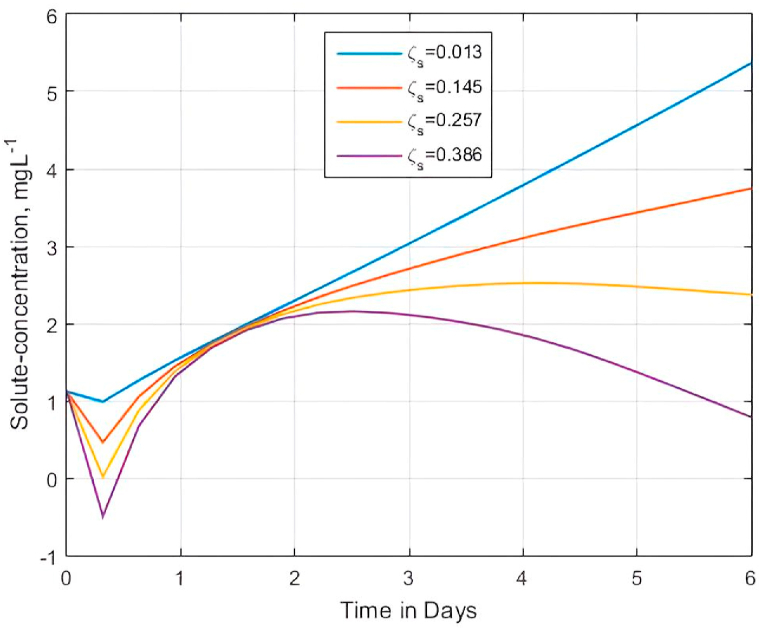
Effect of sink parameter $\zeta_{s}$ on solute transfer profiles for $\varrho_{5}=\left(\zeta_{s}{d^{2}C}_{s}\right)/Ď\left(C_{1}-C_{0}\right)$ when $\rho_{b}=2.69\;g{cm}^{-3}$; pore radius d=1; apparent molecular diffusion $Ď=6.14\times 10^{-4}{\;cm}^{2}s^{-1}$; concentration at the sink $C_{s}=0.001\;g{cm}^{-3}$; initial solute concentration $C_{0}=0.05\;g{cm}^{-3}$; and source concentration $C_{1}=1.25\;g{cm}^{-3}$

Last but not least, we investigated the effect of the sink parameter $\varrho_{5}$ on the behavior of BTCs for the dimensionless solute concentration. In this case, we tried to understand the role of the volumetric flux of water per unit volume of soil matrix $\zeta_{s}\;\left[{day}^{-1}\right]$ on the solute BTCs. To achieve this, we used Eq. (22) to simulate solute transfer BTCs for different values of $\zeta_{s}\;\left[{day}^{-1}\right]$ ($\zeta_{s}$ = 0.013, 0.145, 0.257, and 0.386). Fig. 10 shows the results of the simulations.

In their study, Anwar et al. [127] found that concentration profiles decreased for the increasing values of the sink parameter. Likewise, Fig. 10 confirms that the solute concentration profiles indeed decrease at the increased value of $\zeta_{s}$, consistent with Anwar et al. [127] and others [137]. The physical explanation is that an increase in $\zeta_{s}$ causes the sink coefficient $\varrho_{5}$ to increase. Consequently, the mass transfer coefficient increases with the volumetric flux $\zeta_{s}$ to influence the occurrence of the negative concentration gradient and result in a decrease in the state of concentration in the medium. Going by the earlier made consideration that the role of a sink in the model is mimicked by the plant root uptake activity [110], the results displayed in Fig. 10 suggest the degree to which the current approximate analytical solutions are relevant to other models of water and chemical flow through a 2D porous medium.

(d) Effect of pore water velocity on breakthrough curves

**Fig. 11:**
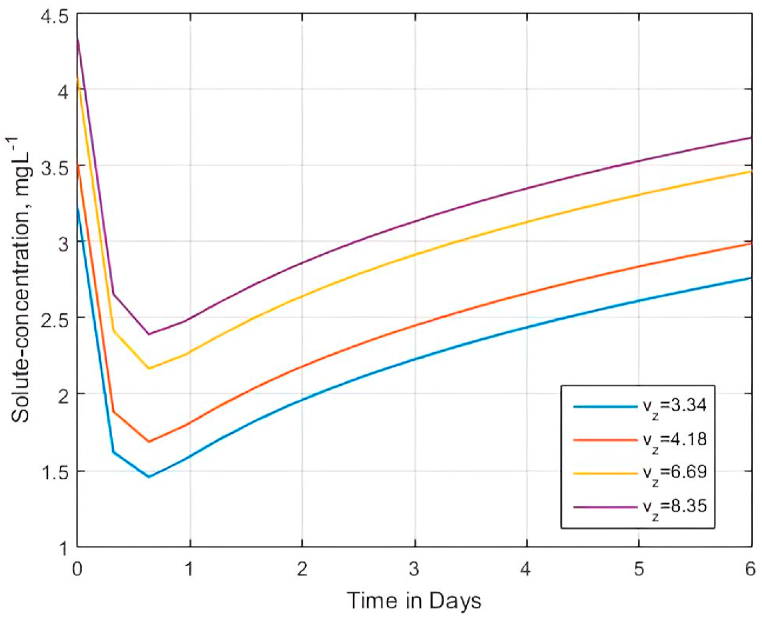
Effect of pore water velocity $v_{z}$ on solute BTCs whenever $q_{w_{x}}=v_{y}=1.0$$cm\;{day}^{-1}$, $C_{0}=0.10\;g{cm}^{-3}$; $C_{1}=0.125\;g{cm}^{-3}$, $\vartheta_{w}$ = 0.21, $R_{m}=1$, $\varrho_{5}=\lambda =1.0\text{,}$Pe = 0.25 and $\alpha_{x}=\alpha_{z}=\vartheta_{w}/Pe$.

To accurately predict the movement of contaminants through soils, it is required to understand the effect of pore water velocity on the solute transport [119]. Also, in some situations, an increase in pore water velocity may lead to an increase in sorption rate coefficient and retardation [138], thereby affecting the movement of the solute concentration. We examined the effect of pore water velocities on solute BTCs using Eq. (22) with average pore water velocities of 3.34, 4.18, 6.69, and 8.35 $cm\;{day}^{-1}$ taken from an experimental study by Zhou and Wang [119]. The physical structure of the model (Fig. 1) entails that infiltration is dominant in the z− direction. Therefore, the investigation was conducted at ${v_{x}=q}_{w_{x}}\neq q_{w_{z}}=v_{z}$ for $q_{w_{x}}<q_{w_{z}}$. For the average pore water velocities of 3.34, 4.18, 6.69, and 8.35 $cm\;{day}^{-1}$ in the z-direction, we conducted simulations at $q_{w_{x}}=1.0$ $cm\;{day}^{-1}$ in the x-direction. Fig. 11 shows the results of the simulations.

Unlike in Ref. [119], we did not investigate the influence of pore water velocity under different (small/large pulse) solute input methods because of the assumption that solute concentration is constant at the source. Nonetheless, Fig. 11 still shows that the pore water velocity affects the solute BTCs because, in the soil matrix, the solute solution moves slowly by convection and diffusion [119]. Thus, the pore water velocity causes a fraction of the solution to flow rapidly through the macrospores [139]. The results are consistent with Shukla et al. [140], who experimentally deduced that for an increase in pore water velocity $v_{m}$, the apparent diffusion coefficient Ď increases. Hence, increased values of pore water velocity aided most of the dissolved solutes to diffuse more rapidly.

Although the computational cost of the present analytical solutions is comparable with respect to numerical solutions but with the advantage of being more precise (see Fig. 3), the present solutions present a few limitations. First, while the derivation process is clear, the limits of integrals obtained from the spatial convolution property were evaluated by adaptive Gauss-Krourod quadrature method while the time integrals were computed using numerical Trapezoidal algorithm during simulations. Given that most of the integral based analytical solutions do not exist in closed form, this shortcoming is understandable [46]. Second, the current 2D analytical solutions are derived on the assumption that both velocity and dispersion coefficients are non-scale dependent. Hence, the solutions represent only the approximate description of the transport and dispersion processes taking place in the soil. Further research is needed for this endeavor, making inference of the detailed summaries of 1D solute transport models with scale-dependent dispersions in Gao et al. [6] and Sanskrityayn et al. [49].

## **5** Summary, Conclusion(s) and future Outlook(s)

### 5.1 Summary and Conclusion(s)

This study began with a rapid review of exact and analytical methods for solving a transport equation described by the ADRE for modeling solute transport in a homogeneous and isotropic porous medium. For each method, we discussed both the advantages and disadvantages. From the literature review, integral-based solution methods are attractive because of their ability to preserve model features. Integral-based methods include the GFM, GITT, Laplace, Fourier transform, Multi-dimensional Laplace transforms, and RITM (which combines Laplace and Fourier transforms). Unlike the GFM, the RITM has been less employed to solve at least 2D solute transport equations despite some advantages. First, there already exists a wide range of tables of results for Laplace and Fourier transform identities. Second, the RITM does not require reducing the original model into a BVP or in-homogeneous diffusive equation, hence maintaining problem originality. Unlike the GFM, some concise expressions for the solute concentration are in closed form. Then, the RITM was applied to obtain approximate analytical solutions of a 2D ADRE coupled with linear sorption, decay, and sink as motivated by the case study of a soil domain with a constant solute point source, located internally but near the top surface and parallel to the vertical flow direction. We have studied the effects of retardation, reaction, sorption, sink, advection, diffusion, and pore water velocity on the solute distribution to validate the application of the derived approximate analytical solutions. A comparison with numerical simulations was performed to verify the accuracy of the approximate analytical results. All simulation results are presented in graphical forms.

The conclusions of the current study are as follows. Distribution of the solute increases for an increase in Peclet number Pe since increasing Pe enhances the power of advection. The first-order chemical reaction rate (${ή}_{w}$) and volumetric water content ($\vartheta_{w}$) significantly affect solute distribution when both are present inside the ‘apparent’ Dank $\ddot{o}$ hler ($\varrho_{3}$) number whenever reaction process is either accelerated ($\vartheta_{w}>0$) or decelerated ($\vartheta_{w}<0$). An increase in retardation ($R_{m}\gg 1$) due to a decrease in volumetric water content ($\vartheta_{w}\ll 1$) or an increase in linear sorption or distribution coefficient $\kappa_{d}$ causes a decrease in distribution profiles. However, the influence of $\kappa_{d}$ is less significant in a non-fractured medium than in a fractured domain [24]. The presence of sink parameter $\zeta_{s}$ in the model for the nutrients, mimicked by the plant-root uptake activity, significantly contributes to the changes of solute concentration in the medium (Fig. 10). An increase in $\zeta_{s}$ causes the solute BTCs in the medium to decrease. Increased pore water velocity aids the solute diffusivity process, thereby increasing concentration state in the medium. As demonstrated by the verification and validation simulations, agreeing with both the theory and the literature, the RITM-based solutions can characterize solute distributions in a (2 + 1) dimensional porous medium. By relaxing the conventional bare surface assumption [15,25], the current proposed solutions have a wide range of usability since we can investigate more model parameters than before.

#### 5.1.1 Future Outlook(s)

• Contrary to the experimental settings of Grisak and Pickens [5], Rahman et al. [4], Gao et al. [6], Dejam [24] and others, the derived approximate analytical solutions are limited to a homogenous porous medium without fracture. It is worthy of extending its applications to the 2D porous medium with fractures.
• It would be interesting to explore new solutions using the GFM and the multi-dimensional Laplace transformation technique [74] and compare with the RITM solutions.
• Solute concentrations calculated by the current approximate analytical solutions have not been compared with measured soil contaminant data. Given an opportunity of working with field data, the solutions are open for further validation using mean-absolute-error or relative mean-square-error (RMSE) [141].
• Lastly, it could be more plausible to relax the assumption of negligible heat effect and consider thermophoresis parameter Ξ≠0 and couple Eq. (7) with heat diffusion equation to formulate a one-way coupled model. Then solve the new equation using a procedure proposed by Bai et al. [142] to study the effect of temperature gradient on moisture and ionic transport in concrete or the influence of moisture gradient on heat distribution [143].

## Declarations

### Author contribution statement

Elias Mwakilama: Performed the experiments; conceived and designed the experiments; Analyzed and interpreted the data; Contributed reagents, materials, analysis tools or data; Wrote the paper.

Duncan Gathungu and Vusi Magagula: conceived and designed the experiments; Analyzed and interpreted the data; contributed reagents, materials, analysis tools or data; Wrote the paper.

### Funding statement

This research did not receive any specific

grant from funding agencies in the public, commercial, or not-for-profit sectors.

### Data availability statement

No data was used for the research described in the article.

## Conflicts of interest

The authors declare no conflict of interest.

### Additional information

No additional information is available for this paper.

## Appendix A

Equation (17) is obtained by, first, setting $\varrho_{5}$ to zero and the velocity components $v_{x}$ and $v_{z}$ to unity in Eq. (16). Then, applying Laplace transform technique on variable t, Eq. (16) with the help of the initial condition in Eq. (9) becomes

A 1 $$\alpha_{z}\frac{\partial^{2}C}{\partial z^{2}}+\alpha_{x}\frac{\partial^{2}C}{\partial x^{2}}-\frac{\partial C}{\partial z}-\frac{\partial C}{\partial x}-\left(s+\lambda \right)C=0$$

where s>0 denotes the Laplace transform parameter and C represents the Laplace transform of C(x,z,t).

Since we assumed that the dominant uniform flow in the z− direction (see Fig. 1(c)), then, taking an infinite Fourier sine transform of Eq. (A 1) with respect to variable x in all but the concentration gradient ∂C/∂x term (an infinite Fourier cosine transform is considered for this term), yields

A 2 $$\alpha_{z}\frac{\partial^{2}F^{s}\left\{C\right\}}{\partial z^{2}}+\alpha_{x}F^{s}\left\{\frac{\partial^{2}C}{\partial x^{2}}\right\}-\frac{\partial F^{s}\left\{C\right\}}{\partial z}-F^{c}\left\{\frac{\partial C}{\partial x}\right\}-\left(s+\lambda \right)F^{s}\left\{C\right\}=0\text{.}$$

Evaluating the resultant second and fourth Fourier integral terms of Eq. (A 2), with the help of the Laplace-Fourier transformed top and bottom boundary conditions, results into

A 3 $$\alpha_{z}\frac{d^{2}}{dz^{2}}\Theta -\frac{d}{dz}\Theta -\left(s+\lambda +\alpha_{x}\omega^{2}+\omega \right)\Theta =\frac{\left(1-\alpha_{x}\omega \right)}{s}\text{,}$$

where Θ denotes the Laplace-Fourier function C(ω,z,s), and |ω|>0 being a Fourier transform parameter. Now, Eq. (A 3) is a second-order non-homogeneous differential equation in the Laplace-Fourier (s−ω) domain. Solving Eq. (A 3) by the method of undetermined coefficient [144] and then using the Laplace-Fourier transformed left and right boundary conditions, we obtain

A 4 $$\Theta =\frac{-2\alpha_{z}q}{Bs\left(B+1\right)}\exp\left(\frac{\left(B+1\right)z}{2\alpha_{z}}\right)-\frac{2\alpha_{z}q}{Bs\left(B-1\right)}\exp\left(\frac{\left(1-B\right)\left(z+1\right)}{2\alpha_{z}}\right)+\frac{4\alpha_{z}q}{s\left(B^{2}-1\right)}\text{,}$$

where $q=\alpha_{x}\omega -1$ and $B^{2}=4\alpha_{x}\left(s+\lambda +\alpha_{x}\omega^{2}+\omega \right)+1$. In Eq. (A 2), $F^{s}$ and $F^{c}$ are respectively, infinite Fourier sine and cosine transforms defined by [28,76]

A 5 $$F^{s}\left(\omega \right)=\int_{0}^{\infty }f\left(x\right)\sin\left(\omega x\right)dx\text{and}\;F^{c}\left(\omega \right)=\int_{0}^{\infty }f\left(x\right)\cos\left(\omega x\right)dx\text{.}$$

Now, we need to invert Eq. (A 4), transforming Θ from the (s−ω) domain to the (t−x) domain. Beginning with the last term of Eq. (A 4), the inverse Laplace transform on s yields

A 6 $$L^{-1}\left\{\frac{4\alpha_{z}q}{s\left(B^{2}-1\right)}\right\}=\frac{q}{\alpha_{x}\left(\omega^{2}+y^{2}\right)}-\frac{q}{\alpha_{x}\left(\omega^{2}+y^{2}\right)}\exp\left(-\alpha_{x}t\left(\omega^{2}+y^{2}\right)\right)\text{,}$$

where y=λαz. The term $\alpha_{x}\left(\omega^{2}+y^{2}\right)$ is obtained by simplifying $\alpha_{x}\omega^{2}+\omega +\lambda$ using the completing the square method and assuming $\alpha_{x}\gg 1$ since by definition $\alpha_{x}\neq 0$.

Now, replacing $\alpha_{x}\omega -1$ with q in Eq. (A 6), and then taking the inverse Fourier transform on ω of Eq. (A 6) leads to

A 7 $$F^{-1}\left[L^{-1}\left\{\frac{4\alpha_{z}q}{s\left(B^{2}-1\right)}\right\}\right]=\frac{\exp\left(-x\sqrt{\frac{\lambda }{\alpha_{x}}}\right)}{2}erfc\left(\frac{x}{2\sqrt{\alpha_{x}t}}-\sqrt{\lambda t}\right)\left\{1-\frac{1}{\sqrt{\lambda \alpha_{x}}}\right\}+\frac{\exp\left(x\sqrt{\frac{\lambda }{\alpha_{x}}}\right)}{2}erfc\left(\frac{x}{2\sqrt{\alpha_{x}t}}+\sqrt{\lambda t}\right)\left\{1+\frac{1}{\sqrt{\lambda \alpha_{x}}}\right\}\text{.}$$

Equation (A 7) is obtained by using the following inverse sine and cosine Fourier identities [60,145], respectively:

A 8 $$F_{s}^{-1}\left\{\frac{\omega }{\omega^{2}+b^{2}}\right\}=\exp\left(-bx\right);\;F_{s}^{-1}\left\{\frac{2\omega }{\omega^{2}+b^{2}}\exp\left(-c^{2}\left(\omega^{2}+b^{2}\right)\right)\right\}=\exp\left(-bx\right)erfc\left(bc-\frac{x}{2c}\right)-\exp\left(bx\right)erfc\left(bc+\frac{x}{2c}\right)\text{,}$$

A 9 $$F_{c}^{-1}\left\{\frac{1}{\omega^{2}+b^{2}}\right\}=\exp\left(\frac{-bx}{b}\right);\;F_{c}^{-1}\left\{\frac{\exp\left(-c^{2}\omega^{2}\right)}{\omega^{2}+b^{2}}\right\}=\frac{\exp\left(b^{2}c^{2}\right)}{2b}\left\{\exp\left(-bx\right)erfc\left(bc-\frac{x}{2c}\right)+\exp\left(bx\right)erfc\left(bc+\frac{x}{2c}\right)\right\}\text{.}$$

Now, for the first term in Eq. (A 4), applying the inverse Laplace transform first, followed by the inverse Fourier transform, gives

A 10 $$F^{-1}\left[L^{-1}\left\{\frac{-2\alpha_{z}q}{Bs\left(B+1\right)}\exp\left(\frac{\left(B+1\right)z}{2\alpha_{z}}\right)\right\}\right]=\int_{-\infty }^{\infty }\frac{1}{\pi {\tau^{\prime }}^{2}}\sqrt{\frac{\alpha_{x}}{2}}\left\{\int_{0}^{\tau^{\prime }}\exp\left(\left(\frac{{-\left(x-\tau^{\prime }\right)}^{2}}{4\alpha_{x}\tau }\right)-\lambda \tau \right)\left\{erfc\left[\frac{\tau -z}{2\sqrt{\alpha_{z}\tau }}\right]\right\}\frac{d\tau }{\sqrt{\tau }}\right\}d\tau^{\prime }+\frac{1}{2\alpha_{z}\sqrt{\pi \alpha_{x}}}\;\int_{0}^{t}\exp\left(\left(\frac{{-\left(x\right)}^{2}}{4\alpha_{x}\tau }\right)-\lambda \tau \right)\left\{erfc\left[\frac{\tau -z}{2\sqrt{\alpha_{z}\tau }}\right]\right\}\frac{d\tau }{\sqrt{\tau }}\text{.}$$

To arrive at Eq. (A 10), we used the identity formula [60].

A 11 $$F^{-1}\left[L^{-1}\left\{\frac{\exp\left(-aA\right)}{sA\left(A+b\right)}\right\}\right]=\frac{\exp\left(ab\right)}{4\alpha_{z}\sqrt{\pi \alpha_{x}}}\;\int_{0}^{t}\exp\left(\left(\frac{{-\left(x\right)}^{2}}{4\alpha_{x}\tau }\right)-\lambda \tau \right)\left\{erfc\left[\frac{a\sqrt{\alpha_{x}}}{\sqrt{\tau }}+\frac{b\sqrt{\tau }}{2\sqrt{\alpha_{x}}}\right]\right\}\frac{d\tau }{\sqrt{\tau }}\text{,}$$

when b=1, $q=\alpha_{x}\omega -1$, and $B^{2}=4\alpha_{x}\left(s+\lambda +\alpha_{x}\omega^{2}+\omega \right)+1$. In addition, we used the following two equations [59,146]:

A 12 $$\int_{0}^{\infty }e^{-sv}\delta \left(v\right)dv=1\text{,}$$

A 13 $$g\left(v\right)*f\left(v\right)=\int_{-\infty }^{\infty }g\left(\tau^{\prime }\right)∙f\left(v-\tau^{\prime }\right)d\tau^{\prime }\text{,}$$

where δ(v) is the Dirac delta function [147] and Eq. (A 13) is the Fourier convolution property. In a similar manner, the second term of Eq. (A 4) can be inversely transformed to

A 14 $$F^{-1}\left[L^{-1}\left\{\frac{2\alpha_{z}q}{Bs\left(B-1\right)}\exp\left(\frac{\left(1-B\right)\left(z+1\right)}{2\alpha_{z}}\right)\right\}\right]=\int_{-\infty }^{\infty }\frac{1}{\pi {\tau^{\prime }}^{2}}\sqrt{\frac{\alpha_{x}}{2}}\left\{\;\int_{0}^{\tau^{\prime }}\exp\left(\left(\frac{{-\left(x-\tau^{\prime }\right)}^{2}}{4\alpha_{z}\tau }\right)-\lambda \tau \right)\left\{erfc\left[\frac{\left(z+1\right)-\tau }{2\sqrt{\alpha_{z}\tau }}\right]\right\}\frac{d\tau }{\sqrt{\tau }}\;\right\}d\tau^{\prime }+\frac{1}{2\alpha_{z}\sqrt{\pi \alpha_{x}}}\;\int_{0}^{t}\exp\left(\left(\frac{{-\left(x\right)}^{2}}{4\alpha_{x}\tau }\right)-\lambda \tau \right)\left\{erfc\left[\frac{\left(z+1\right)-\tau }{2\sqrt{\alpha_{z}\tau }}\right]\right\}\frac{d\tau }{\sqrt{\tau }}\text{,}$$

So that a combination of Eqs. (A 7), (A 10) and (A 14) yields Eq. (17). Following the same procedure, Eq. (22) is obtained, except for the introduction of the Fourier convolution transform formulae.
